# Investigation of Organic Acids in Saffron Stigmas (*Crocus sativus* L.) Extract by Derivatization Method and Determination by GC/MS

**DOI:** 10.3390/molecules25153427

**Published:** 2020-07-28

**Authors:** Laurynas Jarukas, Olga Mykhailenko, Juste Baranauskaite, Mindaugas Marksa, Liudas Ivanauskas

**Affiliations:** 1 Department of Analytical and Toxicological Chemistry, Lithuanian University of Health Sciences, A. Mickeviciaus Str. 9, LT-44307 Kaunas, Lithuania; laurynas.jarukas@lsmuni.lt (L.J.); liudas.ivanauskas@lsmuni.lt (L.I.); 2 Department of Botany, National University of Pharmacy, Valentynivska, Str. 4, 461168 Kharkov, Ukraine; mykhailenko.farm@gmail.com; 3 Department of Pharmaceutical Technology, Faculty of Pharmacy, Yeditepe University Atasehir, Inonu Mah., Kayısdagı Cad., Istanbul 34755, Turkey; baranauskaite.juste@gmail.com

**Keywords:** lactic acid, malic acid, glycolic acid, GC-MS/EI

## Abstract

The beneficial health properties of organic acids make them target compounds in multiple studies. This is the reason why developing a simple and sensitive determination and investigation method of organic acids is a priority. In this study, an effective method has been established for the determination of organic (lactic, glycolic, and malic) acids in saffron stigmas. *N*-(*tert*-butyldimethylsilyl)-*N*-methyltrifluoroacetamide (MTBSTFA) was used as a derivatization reagent in gas chromatography combined with mass spectrometric detection (GC/MS). The saffron stigmas extract was evaporated to dryness with a stream of nitrogen gas. The derivatization procedure: 0.1 g of dried extract was diluted into 0.1 mL of tetrahydrofuran, then 0.1 mL MTBSTFA was orderly and successively added into a vial. Two different techniques were used to obtain the highest amount of organic acid derivatives from saffron stigmas. To the best of our knowledge, this is the first report of the quantitative and qualitative GC/MS detection of organic acids in saffron stigmas using MTBSTFA reagent, also comparing different derivatization conditions, such as time, temperature and the effect of reagent amount on derivatization process. The identification of these derivatives was performed via GC-electron impact ionization mass spectrometry in positive-ion detection mode. Under optimal conditions, excellent linearity for all organic acids was obtained with determination coefficients of R^2^ > 0.9955. The detection limits (LODs) and quantitation limits (LOQs) ranged from 0.317 to 0.410 µg/mL and 0.085 to 1.53 µg/mL, respectively. The results showed that the highest yield of organic acids was conducted by using 0.1 mL of MTBSTFA and derivatization method with a conventional heating process at 130 °C for 90 min. This method has been successfully applied to the quantitative analysis of organic acids in saffron stigmas.

## 1. Introduction

Saffron is considered the world’s most expensive spice and medicinal plant. Besides uses in food, saffron has attracted interest because of its health-promoting properties [1,2]. In addition, saffron stigmas have been proven to have anti-inflammatory, antioxidant, anti-allergic, and antidepressant biological functions [3,4]. These specific properties are considered to be connected to the presence of diverse compounds such as proteins, fats, minerals, sugars, and organic acids [2]. Among these ingredients, organic acids are in a prominent position because not only do they affect the flavor of saffron but also, they have various pharmacological actions [5,6].

Moreover, there is increasing interest in studies examining characteristics of organic acids, searching for positive effects of given compounds on the human body. According to the literature, the short-chain fatty acids, medium-chain fatty acids, and other organic acids have more or less pronounced antimicrobial activity, depending on the concentration of the acids and the bacterial species exposed to the acids [5,6]. It is well known that benzoic and salicylic acids exhibit antibacterial activity, hydroxycinnamic acid, and their derivatives–anti-inflammatory activity, gallic acid is an antimutagenic, anticarcinogenic, and anti-inflammatory agent [7]. Furthermore, succinic acid, acetic acid, citric acid, lactic acid, malic acid, glutamic acid, and their salts promote gastrointestinal absorption of iron [7]. Moreover, citric and malic acids have significant protective effects on the myocardium and act on ischemic lesions, according to a study by Tang et al., where supplying a patients’ diet with these compounds gave significant positive results [8]. Moreover, organic acids play a principal role in maintaining the quality and nutritional value of food. These compounds can be added as acidulants or stabilizers (e.g., citric, ascorbic, benzoic, fumaric, and malic acids).

The quantitative determination of organic acids in such type of samples is of high interest in many industrial and research institutes. However, as the attention on the health benefits of organic acids is increasing, a simple and sensitive method for determining and investigating organic acids is needed [9,10,11,12]. According to the literature, organic acids are weak acids and are only partly dissociated [6]. To increase the stability and solubility of organic acids, different extraction or other chemical processing techniques are needed. The scientific literature describes that one of the most commonly used processes is derivatization, a chemical process for modifying compounds in order to generate new products with better chromatographic properties [13]. Different derivatization techniques can be used; chemical derivatization is usually used for amino acid detection, and it becomes a necessary procedure to transform analytes into derivatives that can be easily isolated, separated, and detected [14,15,16,17]. Gas chromatography is the most widely used and accepted technique for quantitative analysis of derivatization products due to its high resolution, sensitivity, great versatility, and simple sample treatment.

The derivatization parameters were systematically studied. In this study, a simple and sensitive GC/MS method for determination and investigation of organic acids in saffron stigmas after derivatization with *N*-(*tert*-butyldimethylsilyl)-*N*-methyltrifluoroacetamide reagent was presented. The adequacy of the proposed method was estimated in terms of accuracy, linearity, precision, and detection limit. To the best of our knowledge, this is the first report of quantitative and qualitative organic acid GC/MS detection in saffron stigmas with MTBSTFA derivatization reagent, and comparison of different conditions.

## 2. Results and Discussion

### 2.1. Derivatization Solvents and Reagents

When it comes to dealing with highly complex matrices, such as organic acids, it is advisable to use a derivatization process in order to improve parameters of separation, such as volatility, thermal stability, resolution, as well as detection parameters, when gas chromatography is used [18]. During this process, the derivatization reagent plays an important role in the separation and resolution of the analytes [18]. Among the different derivatization reagents for organic acids, silylating agents are the most popular ones, which, moreover, have been proven as excellent reagents for derivatization after extraction. Hence, two silylating agents were tested: *N*-methyl-*N*-(trimethylsilyl) trifluoroacetamide (MSTFA) and MTBSTFA [13,19,20]. This study is intended for increasing knowledge about the behavior and interest of both reagents on the efficiency of organic acid derivatization yield. However, the use of MSTFA yielded an incomplete crocus stigmas extract derivatization of the organic acid, and thus, further studies were performed with MTBSTFA. According to results, significantly 1.2 times higher amount of organic acids (expressed in percentage of the total amount of lactic, malic, and glycolic acids) has been found while using MTBSTFA reagent, and it was selected as a derivatization reagent for further study. Similar results were reported in previous studies [21,22]. Morville et al. evaluated the efficiency of the derivatization process on organic acids’ (glutaric, adipic, and suberic acids) yield when MTBSTFA and MSTFA reagents were used. Results showed that glutaric, adipic and suberic acid yields significantly increased, 1.6, 1.8, and 1.3 times respectively, after using MTBSTFA reagent [21]. This might be explained by the high volatility of MTBSTFA that it did not coelute in the GC system with other peaks and improved parameters of separation, thermal stability and resolution. The MTBSTFA produces dimethyl-*tert*-butylsilyl (TBDMS) derivatives. MTBSTFA-derivatives produce characteristic fragmentation patterns presenting mainly the fragments of [M − 15]^+^ (cleavage of methyl from the molecular ion) and [M − 57]^+^ (cleavage of the *t*-butyl moiety), of which [M − 57]^+^ is generally dominant in the mass spectrum. MSTFA-derivatives yielding trimethylsilyl (TMS) derivatives, mainly show the fragments [M − 15]^+^ (cleavage of a methyl from the molecular ion) and [M − 31]^+^ (cleavage of the trimethylsilyl ether moiety followed by cyclization involving the silyl group). The TBDMS are more stable to hydrolysis than the corresponding TMS derivatives. As demonstrated in previous studies, TBDMS derivatives are formed more easily and have, thus, higher sensitivities (10–100 times) as well as repeatabilities than the corresponding TMS derivatives [22].

**Figure 1:**
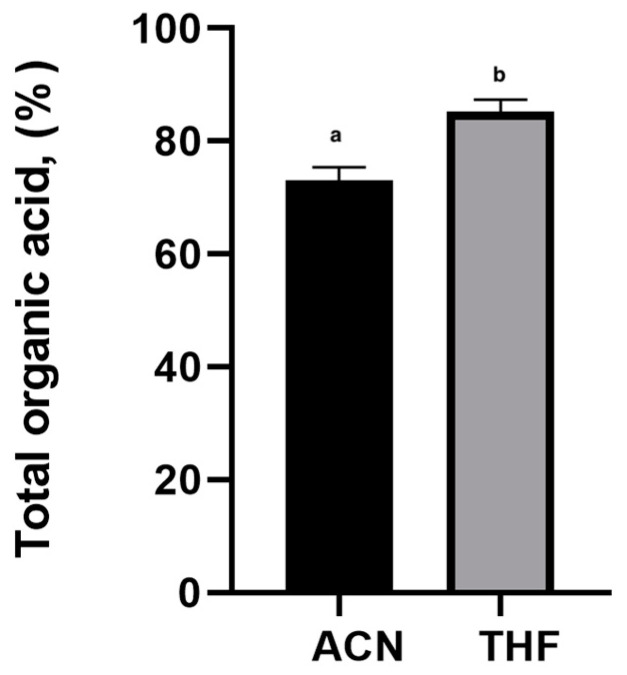
The effect of extraction solvents on the yield of organic acid derivatives (expressed in percentage of the total amount of lactic, malic, and glycolic acids) from Saffron stigmas extract, *n* = 6. Values within columns followed by the same lowercase letter (a, b) differed statistically at *p* < 0.05 (Tukey’s test). Results are expressed as means ± standard error.

The ultimate goal of extraction is the maximization of the yield and coverage of metabolites in a rapid and reproducible way while minimizing enzymatic, chemical, and physical degradation [20]. The derivatization yield of carboxylic acids with MTBSTFA depends on factors including the nature of the solvent in which the analytes are dissolved. The main factors contributing to the increase of the efficiency and the rate of the silylation reaction are the silyl donor ability of the reagent and the ease of silylation of different functional groups in the analyte. The solvent used as a medium and the compounds present or added in the silylation medium may also play a role in derivatization efficiency. The reagent excess is sometimes important for displacing the equilibrium in the desired direction, and usually, an excess up to ten times larger than stoichiometrically needed is used for silylation. The primary purpose was to determine the effect of different solvents (tetrahydrofuran (THF) and acetonitrile (ACN)) on derivatization’s yield of organic acid. During this part of the study, two samples were produced by using derivatization procedure, mentioned above (sample of 0.1 g dried Saffron stigmas extract was diluted into 0.1 mL of extraction solvent, and 0.1 mL derivatization agent (MTBSTFA) was added in sequence; the vial was sealed and oscillated by vortex-mixer for 1 min, then the mixture was placed in glycerol bath allowing it to react at 50 °C for 60 min). Primary investigations revealed that between the ACN and THF samples, significant differences in the organic acid derivate yields (expressed in percentage of the total amount of lactic, malic, and glycolic acids) were obtained (*p* < 0.05; Figure 1).

Moreover, the results showed 1.2 times higher yield of organic acids derivate by using THF as the derivatization solvent in comparison to ACN (*p* < 0.05) (Figure 1). The results could be explained by the sensitivity of the analysis. According to Wittmann et al., by using the THF as extraction solvent increased analysis sensitivity of more polar compounds (lactic acid, oxalic acid, methylcitric acid, 3-hydroxypropionic acid, 3-hydroxyisovaleric acid, kynurenic acid, glycolic acid, orotic acid and quinolinic acid) in comparison with less polar compounds (glycine conjugates) [23]. Hence, the THF was chosen for future experiments.

During this study we compared MSTFA and MTBSTFA in the derivatization efficiency of organic acids. This study is intended for increasing knowledge about the behavior and interest of both reagents. These results clearly demonstrate that solvent plays a significant role in the derivatization procedure. As a matter of fact, MTBSTFA and THF possess the most appropriate derivatization efficiency of the above-mentioned compounds, and they were selected for further studies.

### 2.2. Comparison of the Derivatization Parameters

**Figure 2:**
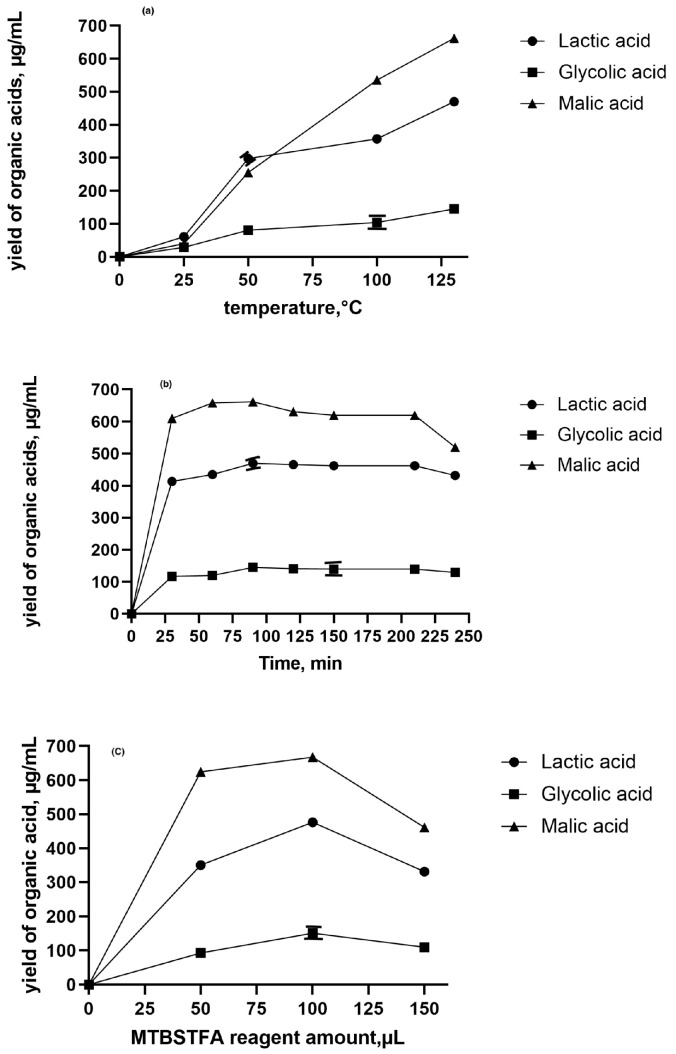
The effect of operation parameters on derivatization reaction: (the yield of lactic acid—62 μg/g, glycolic acid—40 μg/g and malic acid—30 μg/g in the example sample). Temperature (**a**), Time (**b**), derivatization agent (MTBSTFA) (**c**). Results are expressed as means ± standard error (*n* = 6).

Sample preparation is a critical part of every analytical procedure. The increasing demand to determine compounds at low concentrations in complex matrices requires a preliminary step. To achieve the best derivatization efficiency, a variety of important parameters, such as a derivatization temperature, time and amount of MTBSTFA, were optimized. In this study, the saffron stigmas extract was employed to optimize derivatization conditions. The concentration trends of three representative organic acids (lactic, glycolic, and malic acids) relative to different parameters are shown in Figure 2.

The primary purpose was to determine the influence of different temperatures and extraction time on the derivatization yield of investigated organic acids. The results showed that increasing temperature significantly increased the yields of organic acids (Figure 2a; *p* < 0.05). At a derivatization temperature of 130 °C, lactic, glycolic, and malic acid derivative yields significantly increased, 7.7, 16.1, and 5 times, respectively, in comparison with samples prepared at a temperature of 25 °C (Figure 2a). The explanation of such results could be that higher temperature is speculated to enhance derivatization efficiency, by increasing solubility of derivatization reagents and organic acid metabolites [24]. Similarly, Gulberg et al. found that increasing temperatures had an appreciable effect on derivatization efficiency [24]. Moreover, the effect of extraction time (30, 60, 90, 120, 150, 210, and 240 min) on the yield of organic acids derivatization was investigated. The derivatization procedure was carried in the same way as mentioned above. Primary investigations revealed that a prolonged extraction time of 90 min, had no significant influence on derivatization yields of lactic, glycolic and malic acids, extracted from saffron stigmas (*p* > 0.05; Figure 2b). As the reaction time was prolonged, the signal response of organic acid derivatives remained constant. Similarly, Elias and co-authors found that long term silylation-derivatization process was beneficial to stearic acid and glucose-6-phosphate [25]. Therefore, the derivatization reaction between MTBSTFA and organic acids was carried out for 90 min at 130 °C.

To ensure the complete and repeatable derivatization, the desired amount of MTBSTFA reagent was required. The influence of different volumes (50, 100, 150 μL) of MTBSTFA reagent on organic acid yields was optimized. As indicated in Figure 2c, the highest derivatization yield of organic acids was obtained when the reagent amount was 100 μL (*p* < 0.05). However, a decrease in derivatization efficiency was observed when the MTBSTFA reagent amount in the extract increased to 150 μL. According to literature, the most reported amounts of silylation agents for the silylation of polar plant extracts range between 30–125 µL [26]. This is in line with the findings of Koek, who showed that organic acids and sugars need relatively low volumes of the silylating agent [27]. It could be explained as molecular interaction because the reactivity of TMS groups is low to oxygen in organic acids, having a lower number of unshared electrons, higher steric hindrance, and transition state energy [25].

**Figure 3:**
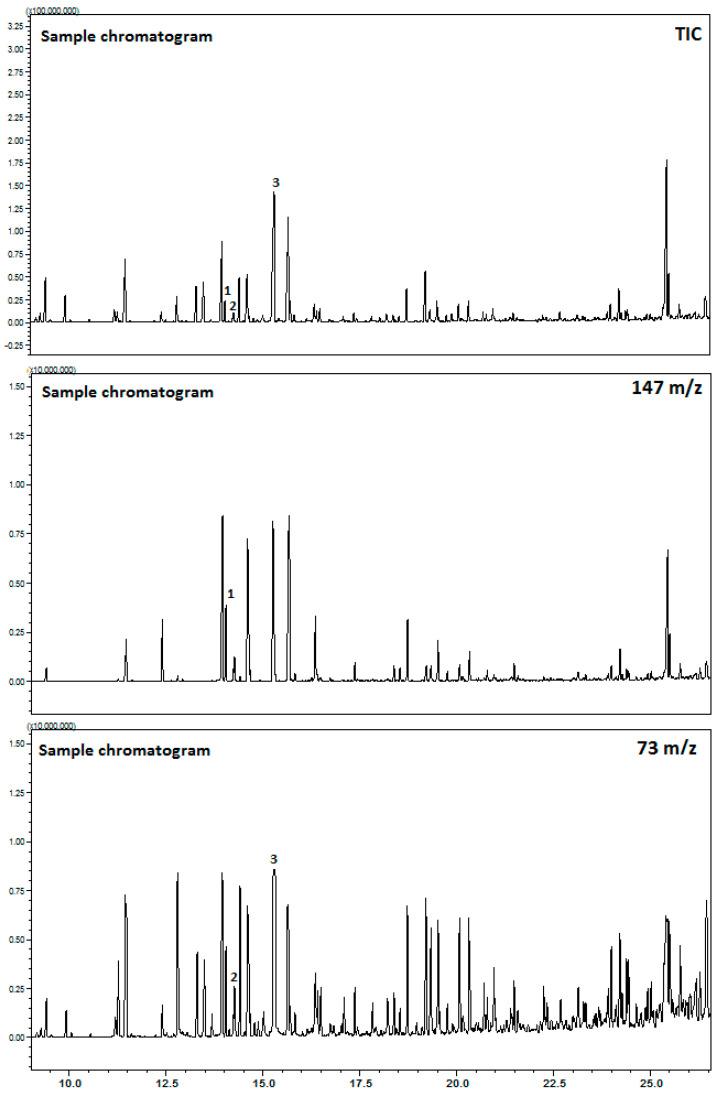
GC/MS chromatograms of standards (1-lactic, 2-glycolic, and 3-malic acids) and organic acid derivatives extracted from Saffron stigmas.

As a final conclusion of this study, when the derivatization temperature was 130 °C, silylation time was 90 min, and the MTBSTFA reagent amount was 100 μL, the highest yield of organic acid derivates in the sample, extracted from the Saffron stigmas was obtained (Table 1). Such a significant shortening of time was achieved by applying high temperature, which allowed to avoid time-consuming sorption of derivatization reagent and time-consuming desorption of analytes, which allowed to reach the high derivatization efficiency. Moreover, the rapid derivatization procedure improved parameters of separation, such as volatility, thermal stability, resolution, as well as detection parameters. For the standard (lactic, glycolic, and malic acids) and saffron stigmas extract, produced by using optimal conditions, chromatograms are shown in Figure 3.

### 2.3. GC/MS Method Validation

The GC method was validated by following the ICH Q2 (R1) guidelines [28]. The developed method was evaluated via the correlation coefficient (R^2^), linear range, detection limit (LOD), quantitative limit (LOQ), accuracy, and precision. The electron impact ionization of lactic, glycolic and malic acids, produced the [M]^+^ ions at 156, 83 and 84 under positive ionization conditions. The product ion spectra ions at *m/z* 147, *m*/*z* 73, and *m*/*z* 73 were produced as the prominent product ions for lactic, glycolic and malic acids (Table 1). The calibration curves of the three organic acids (lactic, glycolic, and malic acids) were established by injecting the standard solutions in the range of 15–242 μg·L^−1^, 12–379 μg·L^−1^ and 12–758 μg·L^−1^, respectively and plotting the average peak areas versus the average concentrations of organic acids based on the data of triplicate measurements. The good linearity response over the tested concentration range was obtained with the developed method for the compounds used as lactic acid standards, having R^2^ > 0.997, as shown in Table 1. The LOD value was 0.153 μg/mL, while the LOQ was 0.317 μg/mL (Table 1), which indicates that the method is sensitive. Moreover, R^2^ values of the glycolic acid standard were higher than 0.996, thus confirming the linearity of the method (Table 1). Thus, the LOD value was 0.101 μg/mL, while the LOQ was 0.41 μg/mL (Table 1), which suggested full capacity for the quantification of the glycolic acid investigated. Furthermore, R^2^ values of the malic acid standard were higher than 0.999, and the LOD value was 0.085 μg/mL, while the LOQ was 0.339 μg/mL (Table 1). According to the described data above, it can be concluded that this method is a reliable tool for the identification and quantification of organic acid in saffron stigmas, conforming to the ICH guidelines.

## 3. Materials and Methods

### 3.1. Materials and Methods

Ultrapure water was obtained in the laboratory using a Milli-Q water purification system (Millipore, Billerica, MA, USA). *N*-(t-butyldimethylsilyl)-*N*-methyltrifluoroacetamide (MTBSTFA) (>99%), *N*-methyl-*N*-(trimethylsilyl) trifluoroacetamide (MSTFA) (>98.5%) tetrahydrofuran, acetonitrile were purchased from Sigma–Aldrich (St. Louis, MO, USA). The GC-equipment was run with helium (purity 5.0) as the carrier gas was purchased from Gazchema (Lithuania). Ethanol (96%) for extraction was purchased from Vilniaus degtinė (Vilnius, Lithuania). Lactic acid (>98%), glycolic acid (>99%), and malic acid (>99%) standards were purchased from Sigma–Aldrich (Co., Birkenhead, UK).

### 3.2. Sample Preparation

Saffron stigma was purchased from Novin Saffron Company, Mashhad, Iran. Prior to the extract preparation, the saffron stigma was dried with a stream of nitrogen gas, then was grounded in a cross beater mill IKA A11 Basic Grinder (IKA Works, Guangzhou, China) and sieved using vibratory sieve shaker AS 200 basic (Retch, UK) equipped with a 125 µm sieve. Then, the powdered sample (1 g) was extracted with 10 mL of 70% (*v*/*v*) methanol-aqua solution in a volumetric flask using an ultrasound bath for 20 min and filtered through a 0.45 μm nylon filter.

### 3.3. Derivatization Procedure

0.1 g of prepared extract solution was evaporated to dryness with a stream of nitrogen gas. Briefly, to a 2 mL ampoule bottle, 0.1 g of dried extract sample was diluted into 0.1 mL of extraction solvent (tetrahydrofuran), and 0.1 mL derivatization agent (MTBSTFA) was added in sequence. The vial was sealed and oscillated by vortex-mixer for 1 min, and then, to allow the mixture to react at room temperature (25 °C) for 60 min, it was placed in glycerol bath. The subsequent solution was transferred to 200 μL insert placed autosampler vials, and 1 μL aliquot was injected into GC-MS system for analysis. Efficiency extraction parameters were evaluated and optimized, including derivatization time, extraction temperature and reagent amount on derivatization. The comparison of chromatographic responses was used to evaluate the extraction efficiency. A similar procedure was used for lactic, glycolic, and malic acid standards. Standards were diluted in the THF, producing a mixture of 150 μmol L^−1^. The same derivatization reaction was applied to this standard mixture, with the exception of the addition of 0.1 mL of THF, because it was already present in the standard mixture.

### 3.4. GC/MS Method

Analyses were performed using a SHIMADZU GC/MS-QP2010nc Ultra chromatography system (coupled to an Electron Ionization (EI) ion source and a single quadrupole MS (Shimadzu Technologies, Kyoto, Japan). A robotic autosampler and a split/splitless injection port were used. Injection port temperature was kept at 250 °C until the end of the analysis. The separation of analytes was carried out on a with Rxi-5 ms (Restek Corporation, Bellefonte, PA, USA, capillary column (30 m long, 0.25 mm outer diameter and 0.25 μm liquid stationary phase thickness) with a liquid stationary phase) 5% diphenyl and 95% polydimethylsiloxane) with helium at a purity of 99.999% as the carrier gas in a constant flow of 1.49 mL/min. The oven temperature was programmed at 75 °C for 5 min, then increased to 290 °C at 10 °C/min and increased to 320 °C at 20 °C/min and kept for 10 min. The total time was 41 min. The temperatures of the MS interface and ion source were set at 280, 200 °C, respectively. The MS was operated in positive mode (electron energy 70 eV). The full-scan acquisition was performed with the mass detection range set at 35–500 *m*/*z* to determine retention times of analytes, optimize oven temperature gradient, and to observe characteristic mass fragments for each compound. Data acquisition and analysis were executed by LabSolution GC/MS (version 5.71) (Shimadzu Corporation). For the identification and quantification of the analytes, single-ion monitoring (SIM) mode was used.

### 3.5. GC/MS Method Validation

The validation of the GC/MS method was performed according to the international guidelines on analytical techniques for the quality control of pharmaceuticals (ICH guidelines) [28]. Method validation was performed to assess linearity, LOD, LOQ, and precision. The calibration curves of the organic acids (lactic, malic and glycolic acids) were established by injecting the standard solutions in the range of 12–758 μg/mL) and plotting the average peak areas versus the average concentrations of organic acids based on the data of triplicate measurements (Table 1). Analytes stock solution was prepared in THF by diluting of the analytical standards to reach a concentration of lactic acid 242 μg/mL, glycolic acid 379 μg/mL, and malic acid 758 μg/mL. Then, the subsequent dilutions were prepared with MilliQ water. The standard solutions (*n* = 3) were prepared at approximate concentrations of lactic acid: 242, 121, 60.5, 30.25, 15.13 μg/mL; glycolic acid: 379, 189.5, 94.75, 47.38, 23.69, 11.84 μg/mL, and malic acid: 758, 379, 189.5, 94.75, 47.38, 23.69, 11.84 μg/mL, due to the wide levels found in saffron samples. The concentrations of the lactic, glycolic and malic acids in each solution was maintained arranged as follows 15.13, 11.84, 11.84 μg/mL, respectively. The LOD and LOQ were calculated at signal-to-noise (S/N) ratios of 3 and 10, respectively. The precision of the method was evaluated by calculating the repeatability (r). The precision of the extraction technique was validated by repeating the extraction procedure with the standard mix solutions six times. An aliquot of each extract was then injected and quantified. The precision of the chromatographic system was tested by checking the %RSD of retention times and peak areas. Six injections were performed each day for three consecutive days.

### 3.6. Statistical Analysis

We used between five and six biological replicates for saffron stigmas extract samples and six technical replicates for standard samples. Each biological replicate consisted of a stigma of 10 plants, resulting in the isolation of 4–8 mg of stigmas. Raw data was assessed using ANOVA statistical testing (specifically one-way analysis of variance) and Tukey’s multiple comparison test. For this purpose, a software package was utilized (Prism v. 5.04, GraphPad Software Inc., La Jolla, CA, USA) with statistical significance being defined as *p* < 0.05. Results were expressed as average ± standard error.

## 4. Conclusions

The method for organic acid analysis in saffron was developed and validated. The method consisted of sample preparation, derivatization, and chromatographic analysis. All steps were extensively studied and optimized for the derivatization procedure. To the best of our knowledge, this is the first report for the GC/MS detection of the amount and types of organic acids in saffron stigmas with MTBSTFA derivatization reagent and comparison of different conditions. The derivatization reaction was rapid, and the maximum yields of organic acids (lactic, glycolic, and malic acids) were observed by using optimal derivatization conditions. The major advantages of optimal conditions led to reach the highest derivatization efficiency of organic acids in only 90 min by using a conventional heating process. The developed method has been successfully applied to the quantification of organic acids in saffron stigmas. This research also shows the interesting agricultural potential of saffron stigmas, in relation to the preparation of certified extracts with a high content of organic acid to be used in the pharmaceutical and nutraceutical area.

## Figures and Tables

**Table 1:** Linearity and sensitivity data for organic acids (lactic, glycolic, malic acids) used as a standard.

Compound	RT	Ion (*m*/*z*)	Linearity Range (µg/mL)	R^2^ *	LOD ^a^ (µg/mL)	LOQ ^a^ (µg/mL)
Lactic acid	14.038	147, 73, 261	15–242	0.9977	0.156	0.317
Glycolic acid	14.265	73, 147, 247	12–379	0.9955	0.101	0.410
Malic acid	15.256	73, 147, 259	12–758	0.9986	0.085	0.339

Experimental conditions as in Section 3.4. * For R^2^ the correlation coefficient. The *p* value was <0.0001 for all calibration curves. ^a^ LOD were based on S/N = 3; LOQ were based on S/N = 10.
